# P-1628. Impact of the BioFire® FilmArray® BCID2 Panel and Antimicrobial Stewardship on Clinical Outcomes in Patients with Antimicrobial Resistant Bacteremia

**DOI:** 10.1093/ofid/ofae631.1794

**Published:** 2025-01-29

**Authors:** Yihan Li, Yanina Dubrovskaya, Justin Siegfried, Arnold Decano, Dana Mazo, Ioannis Zacharioudakis, Kassandra Marsh

**Affiliations:** NYU Langone Health, New York, New York; NYU Langone Health, New York, New York; NYU Langone Health, New York, New York; NYU Langone Health, New York, New York; New York University, New York, NY; New York University, New York, NY; NYU Langone Health, New York, New York

## Abstract

**Background:**

Bloodstream infections (BSIs) carry high rates of morbidity and mortality, with delays in appropriate therapy associated with worse outcomes. Given limited available literature, our objective was to describe the impact of BioFire® FilmArray® Blood Culture Identification 2 (BCID2) panel implementation on clinical outcomes in patients with organisms not covered by empiric regimens.

**Figure:**
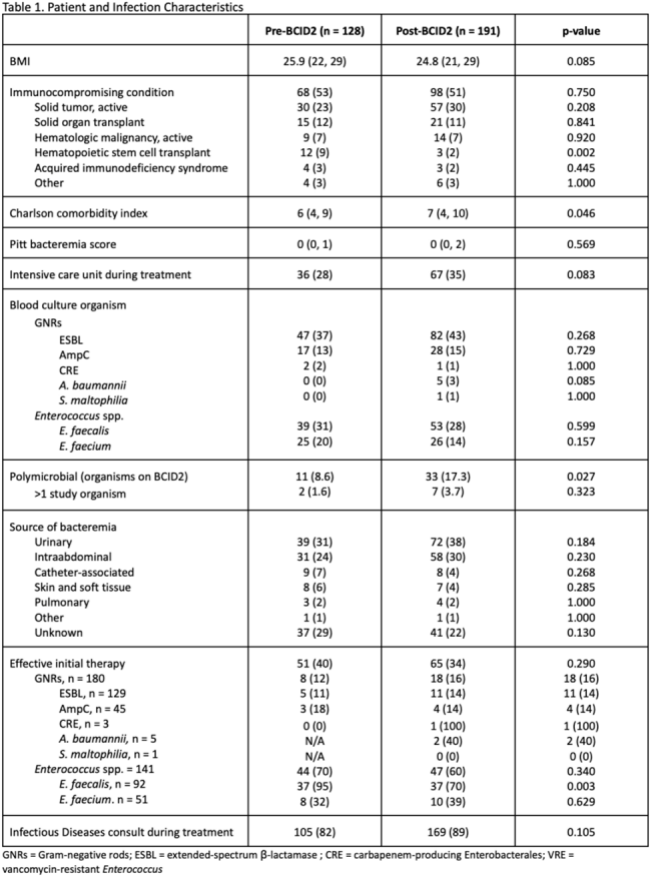

**Methods:**

This was a retrospective review comparing adults with extended-spectrum β-lactamase (ESBL), carbapenemase (CRE), or AmpC-producing Enterobacterales, *Acinetobacter baumannii*, *Stenotrophomonas maltophilia*, or *Enterococcus* species BSIs pre- (7/2020–12/2021) and post- (7/2022–12/2023) BCID2 implementation. Patients were excluded if the initial positive blood culture (BCX) was from an outside facility, had organisms not on the BCID2 panel, or if they died or were discharged prior to BCX results. The primary outcome was time from BCX positivity to initiation of effective therapy. Process measures included pharmacist interventions. Clinical outcomes included recurrence, resistance development, readmission, *Clostridioides difficile* infection, and mortality.

**Figure:**
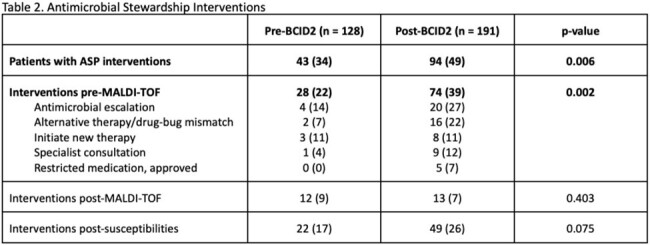

**Results:**

319 patients were included (pre-BCID2 n=128; post-BCID2 n=191), with a median age of 69 years (57-81). The most common infecting organisms were *Enterococcus* spp. (44%) and ESBL Enterobacterales (41%), and the majority were from urinary (35%) or intra-abdominal (28%) sources. Time to effective therapy was significantly shorter post-BCID2 (7 v. 4 hours, p=0.011), particularly among those with ESBL (40 v. 5 hours, p< 0.001), high-risk AmpC-producing organism (41 v. 6 hours, p=0.174), and *E. faecium* infections (17 v. 5 hours, p=0.067). Antimicrobial stewardship pharmacists performed more interventions post-BCID2 (34% v. 49%, p=0.006) with a higher number of interventions performed pre-MALDI-TOF speciation (22% v. 39%, p=0.002). Given low event rates, we were unable to demonstrate clinically meaningful differences in secondary endpoints between groups.

**Figure 1.**
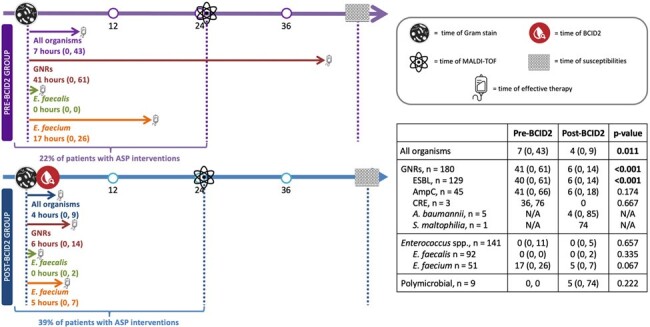
Primary Outcome: Time to Effective Therapy

**Conclusion:**

Implementation of BCID2 in conjunction with antimicrobial stewardship was associated with shorter time to effective therapy. Further studies are needed to understand the impact of this on patient-related outcomes.

**Figure:**
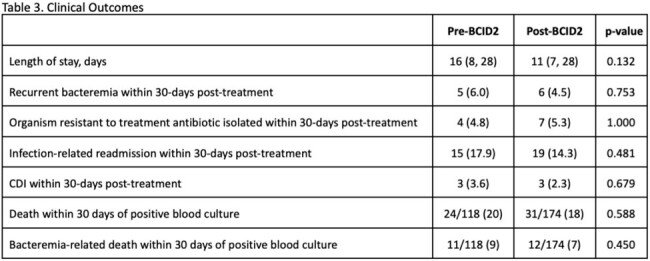

**Disclosures:**

**All Authors**: No reported disclosures

